# An fMRI study of unconditioned responses in post-traumatic stress disorder

**DOI:** 10.1186/2045-5380-1-8

**Published:** 2011-11-01

**Authors:** Clas Linnman, Thomas A Zeffiro, Roger K Pitman, Mohammed R Milad

**Affiliations:** 1 Department of Psychiatry, Harvard Medical School & Massachusetts General Hospital, 149 13th street, Charlestown, MA, USA; 2 Neural Systems Group, Massachusetts General Hospital, 149 13th street, Charlestown, MA, USA

## Abstract

**Background:**

Both fear and pain processing are altered in post-traumatic stress disorder (PTSD), as evidenced by functional neuroimaging studies showing increased amygdala responses to threats, and increased insula, putamen and caudate activity in response to heat pain. Using psychophysiology and functional magnetic resonance imaging, we studied conditioned and unconditioned autonomic and neuronal responses in subjects with PTSD versus trauma-exposed non-PTSD control (TENC) subjects. A design using an electric shock selected by subjects to be 'highly annoying but not painful' as an unconditioned stimulus (US) with partially reinforced cues allowed us to partly disentangle the expectancy- and prediction-error components from sensory components of the unconditioned response.

**Results:**

Whereas responses to the conditioned stimulus (CS) were similar in PTSD and TENC, the former displayed higher putamen, insula, caudate and amygdala responses to the US. Reactivity to the US in the anterior insula correlated with PTSD symptom severity. Functional connectivity analyses using the putamen as a seed region indicated that TENC subjects had increased amygdala-putamen connectivity during US delivery; this connection was disengaged in PTSD.

**Conclusions:**

Our results indicate that although neural processing of fear learning in people with PTSD seems to be comparable with controls, neural responses to unconditioned aversive stimuli in PTSD seem to be increased.

## Background

In classic fear conditioning, an initially neutral, and later conditioned stimulus (CS+) is paired with an aversive unconditioned stimulus (US) such as an electric shock. After pairing, the neutral stimulus is able to elicit fear on its own. Much has been learned about the neural responses induced by the CS+ presentation. In healthy people, conditioned stimuli activate the amygdala, brainstem, insula and parts of anterior cingulate cortex (ACC) [1]. Less is known about changes occurring in response to the presentation of the more biologically relevant US [2]. Responses to a US in healthy subjects are accompanied by increased activity in the brainstem, thalamus, and cingulate, sensory and insular cortices [3-5], structures also known to respond to noxious stimuli [6].

Understanding the neural mechanisms that mediate aversive unconditioned responses may be clinically relevant. Brain regions involved in fear learning, such as the amygdala and the ACC, are implicated in the pathophysiology of several anxiety disorders such as post-traumatic stress disorder (PTSD). Other intrinsically aversive stimuli such as trauma reminders, aversive images and fearful faces also elicit increased responses in several brain regions in PTSD, such as the amygdala and the ACC [7]. The unconditioned response represents a specific exemplar of a more general class of aversive stimuli to which people with PTSD may be more sensitive. In addition, understanding how aversive somatosensory stimuli are processed in PTSD may help to explain why pain processing is altered in this disorder [8], and clarify the high comorbidity between PTSD and chronic pain [9,10]. Recent functional magnetic resonance imaging (fMRI) studies of pain processing in PTSD reported increased insular and putamen reactivity to painful heat [11,12]. However, it is unknown whether such alterations in pain processing are driven by anticipatory or perceptual properties of noxious stimulation, and whether aversive but not overtly painful somatosensory stimuli are also processed differently in PTSD.

In the present study, we examined the neural correlates of responses to electrical shock stimuli in subjects with PTSD and in trauma-exposed non-PTSD (TENC) control subjects. Within an fMRI fear-conditioning paradigm, we examined neuronal and autonomic responses to CS+ and US presentations in a partial-reinforcement paradigm [13]. The US was a 'highly annoying but not painful' electric shock delivered after 62.5% of the CS+ presentations. This experimental design allowed us to compare neural responses to both the CS+ and US, and to the anticipation and unexpected omission of the US (unreinforced CS+ trials). By contrasting time intervals representing delivered versus non-delivered US, the sensory component of a US response may be in part be isolated from its expectancy- and prediction-error related components. Moreover, we related alterations in brain responses elicited by the US to PTSD symptom severity. Lastly, in a *post hoc *analysis, we explored inter-regional functional connectivity differences in US-elicited responses in the regions in which the subjects with PTSD were found to differ significantly from the TENC subjects.

## Methods

The Partners Healthcare System Human Research Committee approved the study, and all subjects gave informed consent.

### Subjects

In total, 23 right-handed people with PTSD and 28 right-handed healthy TENC subjects were recruited from the community. All were administered the Structured Clinical Interview for the *Diagnostic and Statistical Manual*, fourth edition (DSM-IV) [14] to determine PTSD diagnostic status and possible comorbid axis I disorders.

For the TENC group, participants with any current mental disorder were excluded. Subjects with PTSD who had current substance dependence were also excluded, as were subjects who had used any psychotropic medication within 4 weeks before participation (1 year for neuroleptics). Data from four PTSD and four TENC subjects were excluded because of excessive head movement while in the scanner, leaving a total sample size of 19 PTSD and 24 TENC subjects. Data from a subset of these participants have already been reported in studies examining fear extinction [15,16]; however, their responses to the US have not previously been reported.

### Rating scales

The Clinician-Administered PTSD Scale (CAPS) [17], Neuroticism-Extroversion-Openness Personality Inventory-Revised (NEO PI-R) [18], Spielberger State Trait Anxiety Inventory [19], Beck Anxiety Inventory (BAI) [20] and Beck Depression Inventory (BDI) [21] were administered to all participants. These results, along with demographic data, type of trauma, age at first trauma exposure, and current comorbid disorders are shown in Table 1.

**Table 1:** Demographics, comorbidities and type of trauma exposure in the cohort^a ^(significant *P *values in bold).

	**PTSD**^**b**^	**TENC**^**c**^	*P*
**Demographics**			
Gender, M/F	9/10 (n = 19)	11/13 (n = 24)	0.5^d^
Age, ± SD	36 ± 12 (n = 19)	30 ± 12 (n = 24)	0.16
Years of education	15 ± 2 (n = 19)	17 ± 6 (n = 19)	0.1
**Personality**^e^			
NEO neuroticism	27 ± 8 (n = 16)	17 ± 9 (n = 22)	**0.001**
NEO extraversion	22 ± 8 (n = 16)	30 ± 6 (n = 22)	<**0.001**
NEO openness	28 ± 6 (n = 16)	32 ± 5 (n = 22)	**0.04**
NEO agreeableness	29 ± 6 (n = 16)	31 ± 6 (n = 22)	0.3
NEO conscientiousness	33 ± 7 (n = 16)	34 ± 7 (n = 22)	0.66
**Clinical measures**			
CAPS^f ^score	67 ± 24 (n = 19)	8 ± 9 (n = 24)	**< 0.001**
BDI^g^score	21 ± 12 (n = 19)	2 ± 3 (n = 24)	**< 0.001**
BAI^h ^score	19 ± 13 (n = 17)	5 ± 8 (n = 22)	**< 0.001**
Spielberger Trait anxiety	54 ± 12 (n = 18)	35 ± 9 (n = 21)	**< 0.001**
Spielberger State anxiety	44 ± 12 (n = 18)	34 ± 12 (n = 22)	**< 0.001**
Age at trauma, years	24 ± 15 (n = 19)	19 ± 12 (n = 19)	0.23
Time since trauma, years	11 ± 15 (n = 19)	12 ± 12 (n = 19)	0.95
Subjects with childhood^i ^trauma n	9	5	0.10*^d^
**Type of trauma exposure, n**			
Motor vehicle accidents	3	4	1.0^**d**^
Sexual assaults	8	3	**0.04**^**d**^
Physical assaults	5	7	1.0^**d**^
Childhood abuse	6	3	0.15^**d**^
Combat	3	0	0.07^**d**^
Witness to trauma	3	7	0.47^**d**^
**Current comorbidities, n**			
Major depression	5	0	**0.01**^**d**^
Panic disorder	3	0	0.07^**d**^
Alcohol abuse	1	0	0.44^**d**^
Other substance abuse	2	0	0.19^**d**^
Eating disorders	2	0	0.19^**d**^

^a^Not all ratings were available for all participants; numbers are given in brackets. Participants might also have had more than one type of traumatic event or comorbid disorder.

^b^Post-traumatic stress disorder.

^c^Trauma-exposed normal control.

^d^Fisher's exact probability test. All other data were analyzed with Student's *t*-test, two-tailed.

^e^As measured by the Neuroticism-Extroversion-Openness (NEO) Inventory

^f^CAPS, Clinician-Administered PTSD Scale.

^g^BDI, Beck Depression Inventory.

^h^BAI Beck Anxiety Index.

^i^Before the age of 16 years.

### Fear-conditioning procedure

We used a partial-reinforcement classic conditioning paradigm that has previously been described in detail [13]. In the fMRI scanner, each trial began with an image of a room (context) containing an unlit lamp, which was presented for 3 seconds. The lamp was then switched on to show one of three colors (blue, red or yellow). Two of the colors (CS+) were followed by an electric shock (US) in 62.5% of the cases. The third color was never followed by a shock (CS-). The illuminated lamp was presented 32 times, giving a total of 16 safe trials (CS-), 10 CS+ trials followed by the shock, and 6 CS+ trials in which the shock was omitted. Each CS presentation lasted for 6 seconds. Between trials, a black screen was displayed for 12 to 18 seconds. The lamp color sequence was counterbalanced across subjects in pseudo-random order (see Additional file 1 Figure S1).

#### Electric shock

The US consisted of a 500 ms train of 1 ms spikes at 50 Hz delivered to the second and third fingers of the right hand, with currents ranging from 0.2 to 4.0 mA. Before the experiment, the shock current was individually adjusted so that it would be perceived as 'highly annoying but not painful' by the individual patient. Administration of the US immediately followed CS offset.

#### Skin conductance

Skin-conductance responses (SCRs) were measured using a skin-conductance coupler (S71-23; Coulbourn Modular Instruments, Allentown, PA, USA) with 8 mm (sensor diameter) radiolucent Ag/AgCl electrodes (BioPac Systems Inc., Goleta, CA, USA). Electrodes were filled with isotonic paste, and placed on the palm of the subject's left hand. The skin-conductance electrodes were separated by 14 mm. The SCR for each CS trial was calculated by subtracting the mean skin-conductance level 2 seconds before CS onset from the highest skin-conductance level recorded during the following CS duration of 6 seconds. SCR responses during the interval after the US, the omitted (non-delivered) US or the CS- offset were calculated by subtracting the mean skin-conductance level recorded during the first 2 seconds of this interval from the highest skin-conductance level during the ensuing 3 seconds. Because autonomic responses are delayed with respect to their antecedent neural activity [22], this 2-second baseline was more or less uncontaminated by the response to the shock. A representative example of SCR is provided (see Additional file 1 Figure S2).

### Image Acquisition

MRI data were collected using a 3.0 Tesla whole-body MRI system (Trio; Siemens Medical Systems, Iselin, NJ, USA) equipped for echo planar imaging with a 12-channel head coil. Subjects were instructed to lie as still as possible, and head movement was restricted with foam cushions. After an automated scout image was obtained and automated shimming procedures performed, a high-resolution, T1-weighted, three-dimensional, magnetization-prepared rapid acquisition gradient echo (MPRAGE) volume was collected to facilitate spatial normalization and positioning of the subsequent scans. Functional MRI images, sensitive to blood oxygenation level-dependent (BOLD) contrast, were acquired with an interleaved gradient echo T2*-weighted sequence (TR = 3000 ms, TE = 30, flip angle = 90°), collected in 45 coronal oblique slices tilted 30° down from the anterior-posterior commissural line. The voxel size was 3 ± 3 ± 3 mm.

### fMRI data analysis

#### Preprocessing

SPM8 (Wellcome Trust Center for Neuroimaging, http://www.fil.ion.ucl.ac.uk) was used to process all MRI data. Structural images were segmented and spatially normalized to the Montreal Neurological Institute (MNI) 305 T1 template. Functional images were realigned, corrected for slice timing, coregistered with the structural volume, resampled to 2 ± 2 ± 2 mm, normalized into MNI space using parameters obtained from the structural normalization process, and finally smoothed with an 8 mm full width half-maximum Gaussian kernel to reduce spatial noise and to compensate for residual misregistration in the spatial normalization process. High-pass temporal filtering with a cutoff of 128 seconds was included in the first-level statistical model to remove the effects of low-frequency physiological noise. Serial correlations in the fMRI time series caused by aliased biorhythms were estimated using an autoregressive AR(1) model.

#### First-level model

After preprocessing, each subject's functional time series was modeled using a general linear model, with six regressors signifying the condition onsets and durations: the context, onsets of the CS+ and the CS-, the US, the omitted US, and the CS- offset. Movement parameters derived from the realignment step for *x*, *y*, *z *and roll, pitch, and yaw were included in the model to reduce the effects of residual motion-related noise. The experimental effects of interest were identified using a statistical model containing boxcar functions representing each of the six experimental conditions, convolved with the SPM8 canonical hemodynamic response function.

We focused our analysis on the responses to three of the experimental events: 1) the CS+, 2) the US and 3) the omitted US. The CS- cue and the interval following its offset were used as control contrasts. This design allowed us to investigate not only fear-conditioned responses but to also directly contrast brain responses induced by the actual delivery of the US with brain responses induced by the immediate expectancy and then the unexpected omission of the US. Because both the US and omitted US intervals occurred immediately after the offset of the CS+, a combination of anticipatory- and prediction-error components influencing the shock response could be identified in the omitted US contrast, as limited by the temporal resolution of the present fMRI paradigm. Multi-collinearity between regressors may lead to instability of parameter estimation and a consequent reduction in sensitivity. In the present design, the inherent multiple collinearity of the classic conditioning design (a cue followed by a shock) was not problematic for these three contrasts, as the correlations between the regressors were very low (*r*^2 ^< 0.01). However, because of multiple collinearity between regressors identifying cue-induced activations and activity immediately after the termination of the cue (*r*^2 ^= 0.36), we did not examine this effect.

#### Second-level model

First-level contrast images representing the effects of CS+, CS-, US, omitted US and CS- offset were obtained for each subject, and modeled at the second level using a mixed-effects linear model with subject, group and task factors. At the second level, voxel-wise contrasts compared the PTSD group with the TENC group for CS+ versus implicit baseline, CS+ versus CS-, US versus implicit baseline, omitted US versus implicit baseline, and US versus omitted US.

Recent studies of pain responses in PTSD have reported alterations in activity in the putamen, amygdala, hippocampus, cingulate, insula and caudate nucleus [11,12,23]. Based on these studies, we also performed restricted voxel-wise analyses within these regions defined from the Anatomical Automatic Labeling (AAL) library [24,25]. Whole-brain and *a priori *regional activations of more than 10 contiguous voxels, adjusted for multiple comparisons within the search volume thresholded at *P *< 0.05 family-wise error (FEW), corrected for multiple comparisons, are reported.

#### Symptom correlations

First-level contrast images representing the effects of CS+ and US were obtained for all subjects with PTSD, and modeled at the second level using a linear regression model with CAPS scores. Correlated clusters of more than 10 contiguous voxels adjusted for multiple comparisons at *P *< 0.05 FWE are reported. Correlation models such as these have been criticized for overinflated correlation coefficients [26], so we therefore did not plot correlations or report *r *values.

#### *Post hoc *functional connectivity analysis

To further understand alterations in US responses in PTSD, we performed a psychophysiological interaction (PPI) analysis, a technique allowing estimation of how inter-regional BOLD signal correlations vary with respect to an experimental psychological context. Based on the univariate results, we investigated whether the delivery of the US (versus omitted US) modulated the inter-regional functional connectivity between seed regions and targets elsewhere in the brain differently in PTSD and TENC subjects. Four seed regions (the left and right putamen, the left middle frontal gyrus, and the left parahippocampal gyrus) were chosen based on the peak observed hyperactivation to the US of these regions in the PTSD group. We contrasted inter-regional influences of the seeds during the US interval with those during the omitted US interval to elucidate how the delivery of the US modulated seed functional connectivity to other brain regions. The corresponding PPI design matrix had three columns: the interaction between the experimental manipulation and seed time series, the main effect of experimental manipulation (US or omitted US), and the physiological effect (hemodynamically deconvolved seed region time series). In accordance with the task-related univariate analysis, we also included six estimated movement parameters to account for any residual effects due to inter-scan head motion. The voxel-wise regression of voxel time series from the entire brain on the three predictor variables resulted in a first-level PPI contrast for each seed region in every subject. Subsequently, these PPI contrast images were modeled in a second-level group analysis using a mixed-effects repeated-measures linear model with subjects, diagnosis and seed region as factors. This second-level analysis resulted in a statistical parametrical map of regions that displayed differences between TENC and PTSD subjects in alterations of seed functional connectivity depending on the presence or absence of the US. Clusters of more than 10 contiguous voxels adjusted for multiple comparisons at *P *< 0.05 FWE are reported.

## Results

### Shock levels and skin-conductance responses

PTSD and TENC subjects chose similar shock levels and displayed similar levels of autonomic skin-conductance responses to the CS+ and the US. Subjects with PTSD displayed slightly higher skin-conductance responses to the non-reinforced CS- cue, but the magnitude of responses to the CS- was generally low (Table 2; see Additional file 1 Figure S3. Figure S4). There were no significant differences between PTSD and TENC subjects in brain activations to the CS+, the CS- or to the contrast CS+ versus CS-.

**Table 2:** Shock levels and skin-conductance responses (in square root microSiemens, μS^1/2^) to the conditioned stimulus, the unconditioned stimulus (US) and the omitted US ± standard error (significant *P *values in bold).

	**PTSD**^**a**^	**TENC**^**b**^	*P*
Shock level, mA	2.13 ± 0.16	2.20 ± 0.18	0.77
CS^c^+ response, μS^1/2^	0.27 ± 0.08	0.17 ± 0.04	0.24
CS- response, μS^1/2^	0.08 ± 0.04	-0.01 ± 0.04	**0.04**
Unconditioned response, μS^1/2^	0.79 ± 0.11	0.71 ± 0.07	0.56
Omitted US response, μS^1/2^	0.26 ± 0.05	0.21 ± 0.03	0.36

^a^Post-traumatic stress disorder.

^b^Trauma-exposed normal control.

^c^CS, conditional stimulus (positive or negative).

### Brain responses to the unconditioned stimuli

**Figure 1:**
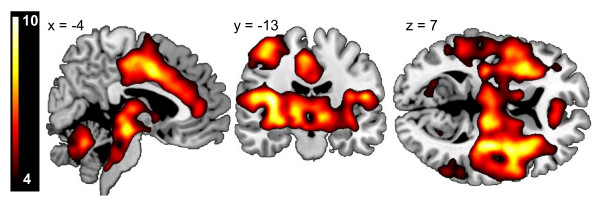
**Main effect of unconditioned responses**. Unconditioned stimulus (US) versus negative conditioned stimulus (CS)- offset in subjects with post-traumatic stress disorder (PTSD) and trauma-exposed non-PTSD control (TENC) subjects. Although the US was 'highly annoying' rather than painful, it elicited massive neuronal responses in classic pain regions including the brainstem, the thalamus, the contralateral sensory cortices, the insula and the middle cingulate. Activations are displayed at a threshold of T > 4 on a template magnetic resonance imaging (MRI) scan.

**Figure 2:**
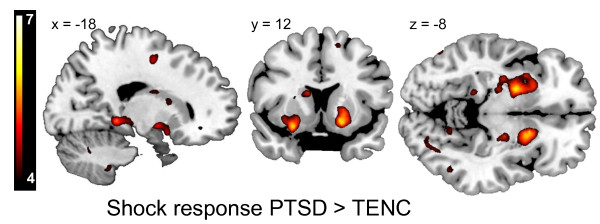
**Increased putamen unconditioned stimulus (US) responses in subjects with post-traumatic stress disorder (PTSD)**. US responses in PTSD minus trauma-exposed non-PTSD control (TENC) displayed at T > 4 on a template magnetic resonance imaging (MRI) scan. The bilateral putamen displayed the most prominent hyperactivation in PTSD, no region displayed hypoactivation in PTSD.

For all subjects, the delivery of the US-elicited responses in the primary and secondary somatosensory cortices, insula, cingulate gyrus, thalamus and brainstem (Figure 1). In the whole-brain between-group contrast, people with PTSD exhibited higher BOLD reactivity to the US in the bilateral putamen, left middle frontal gyrus and left parahippocampal gyrus. Within the *a priori *regions of interest (ROI), subjects with PTSD displayed additional significant hyper-reactivity in the bilateral amygdala, left hippocampus, right dorsal ACC, bilateral posterior insula, left anterior insula, and bilateral caudate and putamen. No regions displayed lower reactivity in the PTSD group (Table 3; Figure 2).

**Table 3:** Univariate analysis results

Contrast **ROI**^**a**^	MNI^b^, peak *x*, *y*, *z*	*Z*-score	Size, voxels	**Cluster *P *value, FWE**^**c**^	Region
**US**^**d**^					
**PTSD^e ^> TENC**^**f**^					
Whole brain	**-22, 0, -6**	**5.51**	**111**	**< 0.0001**	**Left putamen**
	(**-**24, 10, -14)	**-**5.47			
	(**-**32, 2, **-**4)	**-**4.93			
	**18, 10 -12**	**5.47**	**90**	**< 0.0001**	**Right putamen**
	(20, 10, -4)	**-**5.13			
	**-22, -4, 48**	**5.25**	**19**	**0.003**	**Left middle frontal gyrus**
	**-18, -38, -4**	**5.07**	**11**	**0.007**	**Left parahippocampal gyrus**
Putamen	**16, 12, -10**	**5.34**	**359**	**< 0.0001**	**Right ventral putamen**
	(30, -20, 6)	**-**3.79			
	(28, -2, -6)	**-**3.73			
	**-26, 2, -4**	**5.11**	**840**	**< 0.0001**	**Left putamen**
	(**-**22, 8, **-**10)	**-**4.88			
	(**-**32, **-**12, **-**6)	**-**4.75			
Amygdala	**-22, 6, -16**	**4.3**	**48**	**0.01**	**Left amygdala**
	**24, 2, -16**	**4.27**	**81**	**0.005**	**Right amygdala**
Hippocampus	**-18, -32, 4**	**4.43**	**36**	**0.029**	**Left hippocampus**
	(-22, -24, -8)	-3.74			
	**-26, -34, 8**	**3.79**	**18**	**0.028**	**Left hippocampus**
	(**-**14, **-**32, 10)	**-**3.52			
	**-26, -12, -12**	**3.69**	**25**	**0.042**	**Left hippocampus**
	(**-**20, **-**4, 12)	**-**3.62			
Cingulate	**8, 12, 42**	**3.8**	**19**	**0.013**	**Right dorsal acc**
Insula	**34, -20, 4**	**4.97**	**36**	**0.006**	**Right posterior insula**
	**-26, 14, -16**	**5.05**	**28**	**0.008**	**Left piriform insula**
	**-34, -6, 16**	**4.14**	**13**	**0.017**	**Left middle insula**
	**-36, -22, 4**	**4.08**	**17**	**0.014**	**Left posterior insula**
Caudate	**-14, 16, -8**	**4.2**	**67**	**0.006**	**Right caudate**
	(16, 18, 0)	**-**3.81			
	**16, 0, 16**	**3.86**	**24**	**0.021**	**Right caudate**
	**-12, 10, 14**	**4.41**	**297**	**0.001**	**Left caudate body**
	(**-**18, **-**2, 22)	**-**4.16			
**TENC > PTSD**	No significant differences in whole brain or ROI				
**Omitted US**					
**PTSD > TENC**					
Whole brain	No significant differences in whole brain				
Hippocampus	**22, -10, -20**	**3.77**	**54**	**0.009**	**Right anterior hippocampus**
	(30, **-**10, **-**18)	**-**3.46			
**TENC > PTSD**	No significant differences in whole brain or ROI				
**US minus omitted US**					
**PTSD > TENC**					
Whole brain	No significant differences in whole brain				
Putamen	**20, 12, -10**	**3.89**	**69**	**0.003**	**Right ventral putamen**
	**20, 0, 12**	**3.48**	**13**	**0.032**	
Insula	**-28, 16, -18**	**4.05**	**52**	**0.016**	**Left orbital insula**

Clusters are in **bold**, with sub-peaks in brackets.

^a^Region of interest

^b^Montreal Neurological Institute.

^c^Family-wise error.

^d^US, unconditioned stimulus

^e^Post-traumatic stress disorder.

^f^Trauma-exposed normal control.

### Brain responses to the omitted US

During those trials in which the shock was unexpectedly omitted, at a time point when immediate anticipation, prediction error and relief from not getting shocked are intermingled in the hemodynamic response measurable in the current fMRI experiment, subjects with PTSD displayed significantly greater activity in hippocampus. This difference was driven by a lack of deactivation of the hippocampus to the omitted US in the PTSD group. No other significant between-group differences were noted in this analysis (Table 3).

### Brain responses to US versus the omitted US

To partially separate sensory components of the US from anticipatory, prediction error and relief components, we contrasted US versus omitted US in PTSD versus TENC subjects (two-way ANOVA). Within the *a priori *ROI, this contrast showed significantly greater reactivity of the putamen and the anterior insula in the PTSD group (Table 3).

### Symptom correlations

**Figure 3:**
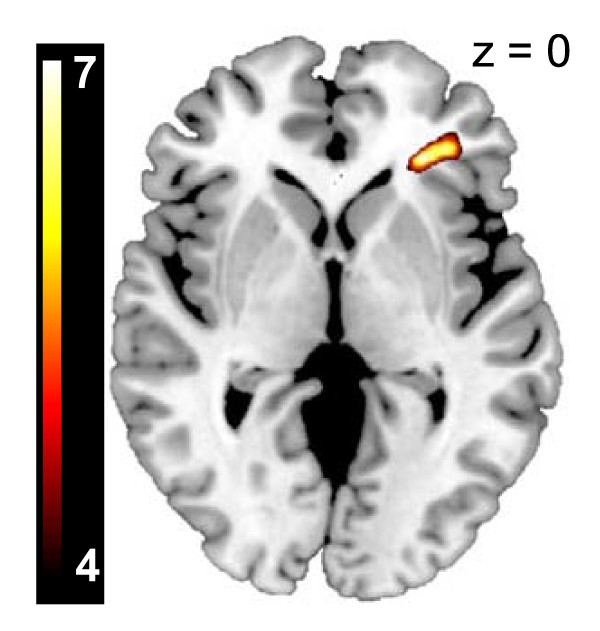
**Post-traumatic stress disorder (PTSD) symptom severity correlates with the magnitude of unconditioned stimulus (US) reactivity**. Positive correlations between blood oxygenation level-dependent (BOLD) US signal in the right anterior insula/inferior frontal gyrus and Clinician-Administered PTSD Scale (CAPS) score in the 19 subjects with PTSD investigated. The correlation map is displayed at a threshold of T > 4 on a template magnetic resonance imaging (MRI) scan.

We found a significant correlation between PTSD symptoms (CAPS scores) and US-induced BOLD activation in the right inferior frontal gyrus/anterior insula (MNI_xyz _= 28, 34, 0, cluster size = 329 voxels, *Z *= 7.93, P_FWE corrected _= 0.002) (Figure 3).

### Functional connectivity of hyperactivated regions

To further elucidate the findings from the univariate analysis, we performed *post hoc *functional connectivity analyses on the regions displaying higher BOLD response to the US in PTSD versus TENC subjects. We chose the US and the omitted US as the conditions possibly inducing altered functional connectivity. In other words, we investigated how the functional connectivity of the seed regions was different during the delivery of the US compared with the omitted delivery of the US. Individual seeds of the putamen, middle frontal gyrus and parahippocampal gyrus were extracted and used in a psychophysiological interaction (PPI), that is, connectivity, analysis.

#### Left putamen

**Figure 4:**
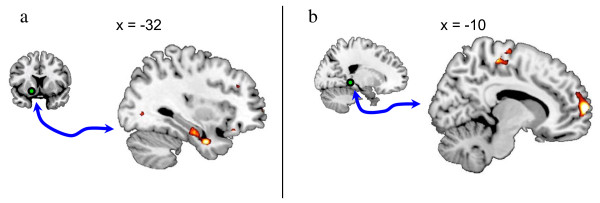
**Psychophysiological interaction (PPI) analysis**. The PPI analysis contrasted the unconditioned stimulus (US) versus omitted US, modeling the change in regional coupling between the seed induced by the delivery of the US. The green sphere indicates the location of **(A) **the putamen seed and **(B) **the parahippocampal seed. Group differences (trauma-exposed non-PTSD control (TENC) > PTSD) in psychophysiological interaction effects are displayed at a threshold of T > 3 on a template magnetic resonance imaging (MRI) scan.

The left putamen displayed the most prominent hyperactivation in the PTSD group, consistent with previous studies using painful levels of heat [11,12], but its role in PTSD is less well described. We thus chose the putamen region *post hoc *to perform a functional connectivity analysis. The seed consisted of a 6 mm sphere centrered at the peak location (MNI_xyz _= -22, 0, -6). When comparing PPI effects of the left putamen between PTSD and TENC subjects, there were significant differences in functional connectivity shifts. The TENC group showed a a more positive PPI effect between the left putamen and the left temporal lobe including the amygdala. In other words, the shock delivery led to a larger increase in functional connectivity between the putamen and the amygdala in the TENC group as compared to connectivity shifts in the PTSD group (Table 4; Figure 4a).

**Table 4:** Psychophysiological interaction analysis

Seed	MNI^a^, peak *x*, *y*, *z*	*Z*-score	Size, voxels	**Cluster *P *value, FWE**^**b**^	**Region**^**c**^
**Left putamen**					
TENC^d ^> PTSD^e^	**-44, -12, -16**	**4.65**	**295**	**0.01**	**Left temporal lobe**
	(-32, 0, -30)	4.01			Left parahippocampal lobe/amygdala
	(-30, -10, -22)	3.72			Left parahippocampal gyrus
PTSD > TENC	No significant differences				
**Right putamen**					
TENC > PTSD	**-30, 0, -32**	**4.54**	**248**	**0.019**	**Left temporal lobe wm/amygdala**
	(-48, 8, -30)	3.97			Left middle temporal gyrus
	(-56, 4, -26)	3.44			Left middle temporal gyrus
PTSD > TENC	No significant differences				
**Left middle frontal gyrus**					
TENC > PTSD	No significant differences				
PTSD > TENC	No significant differences				
**Left parahippocampal gyrus**					
TENC > PTSD	**44, -34, 42**	**4.45**	**449**	**0.001**	**Right inferior parietal lobule**
	48, -38, 50	-4.27			Right inferior parietal lobule
	52, -26, 50	-3.77			Right postcentral gyrus
	-**10, 66, 10**	**4.42**	**263**	**0.017**	**Left medial frontal gyrus**
	(-14, 62, 0)	-4.06			Left medial frontal gyrus
	(-8, 62, 18)	-3.45			Left medial frontal gyrus
	**34**, -**20, 68**	**4.37**	**487**	**0.001**	**Right precentral gyrus**
	(34, -16, 46)	-4.14			Right precentral gyrus
	(22, -16, 74)	-4.12			Right precentral gyrus
PTSD > TENC	No significant differences				

**^a^**Montreal Neurological Institute.

**^b^**Family-wise error.

**^c^**Regions were identified based on nearest gray matter.

^d^Post-traumatic stress disorder.

^e^Trauma-exposed normal control.

#### Right putamen

The right putamen seed (a 6 mm sphere) was centred at the peak location (MNI_xyz _= 18, 10, -12). Contrasting PPI effects of the right putamen between PTSD and TENC subjects showed differences in functional connectivity shifts similar to the left putamen seed, with a more positive PPI effect in the TENC group between the right putamen and the left temporal lobe including the amygdala, that is, indicating a larger increase in functional connectivity between these two regions at the shock delivery in the TENC group compared with the PTSD group (Table 4).

#### Left middle frontal gyrus

The left middle frontal gyrus seed (a 6 mm sphere) was centred at the peak location (MNI_xyz _= -22, -4, 48). Contrasting PPI effects of the left middle frontal gyrus between PTSD and TENC subjects showed no significant differences in PPI effects.

#### Left parahippocampal gyrus

The left parahippocampal gyrus seed (a 6 mm sphere) was centred at the peak location (MNI_xyz _= -18, -38, -4). Contrasting PPI effects of the left parahippocampal gyrus between PTSD and TENC subjects showed differences in functional connectivity shifts, with a more positive PPI effect in the TENC group between the left parahippocampal gyrus and right inferior parietal lobule, and the left medial frontal gyrus and the right precentral gyrus, that is, indicating a larger increase in functional connectivity between the seed and these three regions at the delivery of the shock in the TENC group compared with the PTSD group (Table 4 and Figure 4b).

## Discussion

The predictable and 'annoying' shock (the unconditioned stimuli (US)) elicited activation in several brain regions associated with pain processing [6], suggesting that stimuli may not need to be perceived as overtly painful to activate the so called 'pain matrix'. Subjects with PTSD had significantly higher BOLD reactivity in the putamen, middle frontal gyrus and parahippocampal gyrus compared with the TENC subjects. The ROI analysis further revealed the amygdala, hippocampus, dorsal ACC, insula and caudate nucleus to be hyperactive in the PTSD US response. These findings are consistent with previous studies indicating higher putamen and insula reactivity to heat pain in PTSD [11,12], and increased caudate signaling to heat pain after traumatic memory induction [23]. Subjects with PTSD displayed hyperactivation of the amygdala and dorsal ACC to the US, findings that are consistent with several previous studies suggesting amygdala and cingulate involvement in processing aversive stimuli [5,27-29] and hyperactivation in PTSD [30]. Thus, a highly annoying but not overtly painful electrical shock can be used to replicate previous studies indicating alterations in PTSD pain processing. The benefits of using experimental stimuli that are not overtly painful are obvious.

The CS+ led to comparable increases in skin conductance in both the PTSD and the TENC group, suggesting no differences in the fear induced by the conditioned cues. Moreover, there were no differences in the neural response to the CS+, replicating behavioral studies that suggested intact fear learning in PTSD [31-35], but other studies have disagreed [36-38]. Autonomic responses to the US were of comparable magnitude in PTSD and TENC subjects, as has been previously reported [34,36].

Subjects with PTSD displayed increased putamen reactivity to the US, an effect that was not observed after the omitted US. Moreover, the putamen hyperactivation to the US in the PTSD group remained significant when controlling for responses to the omitted US, (PTSD (US vs. omitted US) vs. TENC (US vs. omitted US); Table 3). We therefore interpret the putamen reactivity as driven mostly by sensation rather than alterations in anticipation of the aversive stimulus or a signal of error prediction. It has been shown that learning of the CS-US relationship allows healthy people to engage in endogenous pain inhibition [39-41]. When endogenous opioid neurotransmission is blocked with naloxone in healthy subjects, a moderately painful US leads to increased putamen and insular responses [42], similar to the present findings. There is evidence that people with PTSD have an altered opioidergic system [43-45], and the findings in the present study seem to support this.

With regard to the functional connectivity results, two major effects were seen when comparing connectivity at the omitted US and the delivered US: TENC subjects (compared with subjects with PTSD), displayed a larger increase in putamen to temporal lobe/amygdala connectivity at the delivered US compared with the omitted US. Moreover, TENC subjects (compared with subjects with PTSD) displayed an increase in parahippocampal gyrus connectivity to the inferior parietal lobule, the precentral gyrus and the medial frontal gyrus (Figure 4a, b). We speculate that the PPI results may be indicative of an altered network processing of aversive stimuli PTSD, but further studies are needed to elucidate how the observed network connectivity changes might relate to the emotional and functional response to aversive stimuli in PTSD.

Higher right anterior insula US reactivity was correlated with higher PTSD symptom severity on the CAPS. This result is consistent with findings of Mickleborough and colleagues [23], who reported a positive correlation between CAPS scores and insula signaling during heat pain after traumatic memory activation. Dickie and colleagues [46] reported a positive CAPS-insula correlation with remembered fearful faces. Felmingham and colleagues [47] reported a positive correlation between CAPS and insula activation to masked fear faces. Thus, this relationship seems to be consistent whether the stimuli are perceived as painful, highly annoying but not painful, encoded as aversive, or subliminally aversive. However, Strigo and colleagues [12] found the rostral anterior insula reactivity to painful stimulation to be negatively correlated to CAPS avoidance scores. One possible explanation for this discrepancy is that in the Strigo experiment, pain stimulation was delivered without a predictive cue, whereas the study of Mickleborough and the present study entailed a predictive cue.

The putamen is best known as a motor output region [48], but both nociception [49,50] and emotion [51] are processed in the putamen and the adjacent nucleus accumbens [52,53]. Moreover, the putamen is structurally and functionally connected to the medial orbitofrontal cortex and the amygdala [54], two regions implicated in PTSD. The observed increased putamen activation in PTSD corresponds to regions known to receive projections from the dorsal anterior cingulate and orbital cortex [55]. Altered basal ganglia function has previously been reported in PTSD for other paradigms: putamen blood flow and reactivity after script-driven imagery in PTSD has been correlated to aspects of flashback intensity and dissociative states [23,56], caudate and putamen volume are decreased in people with PTSD with headaches [57], and people with PTSD display diminished caudate and putamen signal to reward [58]. Thus, future studies could be aimed at elucidating PTSD alterations in both approach and avoidance behaviors involving corticostriatal circuitry [55,59].

### Limitations and alternate interpretations

We have interpreted the neuronal responses and connectivity modulations related to the omitted US as reflecting immediate expectancy ('I am going to get shocked right now'). It could be argued, however, that these results also represent a prediction error and relief signal ('Oh, I guess I did not get shocked'). Our experimental design did not allow us to distinguish between these two responses, thus further studies with higher temporal resolution should be designed and conducted to further examine this point.

## Conclusions

Previous studies have implicated the putamen and insula in altered pain processing in PTSD. The present study extends to this literature by showing that the functional activation of these brain regions is also altered in response to highly annoying but not overtly painful stimuli, and that such alterations are likely to be driven by sensory aspects of the aversive stimulus rather than by expectancy- and prediction-error components.

## List of abbreviations

ACC: anterior cingulate cortex; BAI: Beck Anxiety Inventory; BDI: Beck Depression Inventory; CAPS: Clinician-Given PTSD Scale; CS conditioned stimulus; fMRI functional magnetic resonance imaging; FWE: family-wise error corrected for multiple comparisons; PPI: psychophysiological interaction; PTSD: post-traumatic stress disorder; SCR: skin-conductance response; STAI: Spielberger State Trait Anxiety Inventory; TENC: trauma-exposed non-PTSD controls; US: unconditioned stimulus.

## Competing interests

The authors declare that they have no competing interests.

## Authors' contributions

CL collected and analyzed the data, and wrote the manuscript. TAZ contributed to data analysis and to the final version of the manuscript. RKP participated in experimental design and the final version of the manuscript. MRM designed the study, collected data and contributed to the final version of the manuscript. All authors read and approved the final manuscript.

## Supplementary Material

Additional file 1 **Figure S1 Paradigm design and timing**. Figure S2 Skin conductance responses within three different trials in one representative subject. Figure S3 Skin conductance responses to the conditioned stimulus. Figure S4 Skin conductance responses to the unconditioned stimulus. Figure S5 Skin conductance responses to the omitted unconditioned stimulus. Click here for file
