# Supplementary material for: An fMRI study of unconditioned responses in post-traumatic stress disorder
Source: Biol Mood Anxiety Disord. 2011 Nov 1;1:8. doi: 10.1186/2045-5380-1-8 (PMC3384234; doi:10.1186/2045-5380-1-8)
Supplement: Additional file 1 — Figure S1 Paradigm design and timing. Figure S2 Skin conductance responses within three different trials in one representative subject. Figure S3 Skin conductance responses to the conditioned stimulus. Figure S4 Skin conductance responses to the unconditioned stimulus. Figure S5 Skin conductance responses to the omitted unconditioned stimulus. [file 2045-5380-1-8-S1.PPT]

## Slide 1
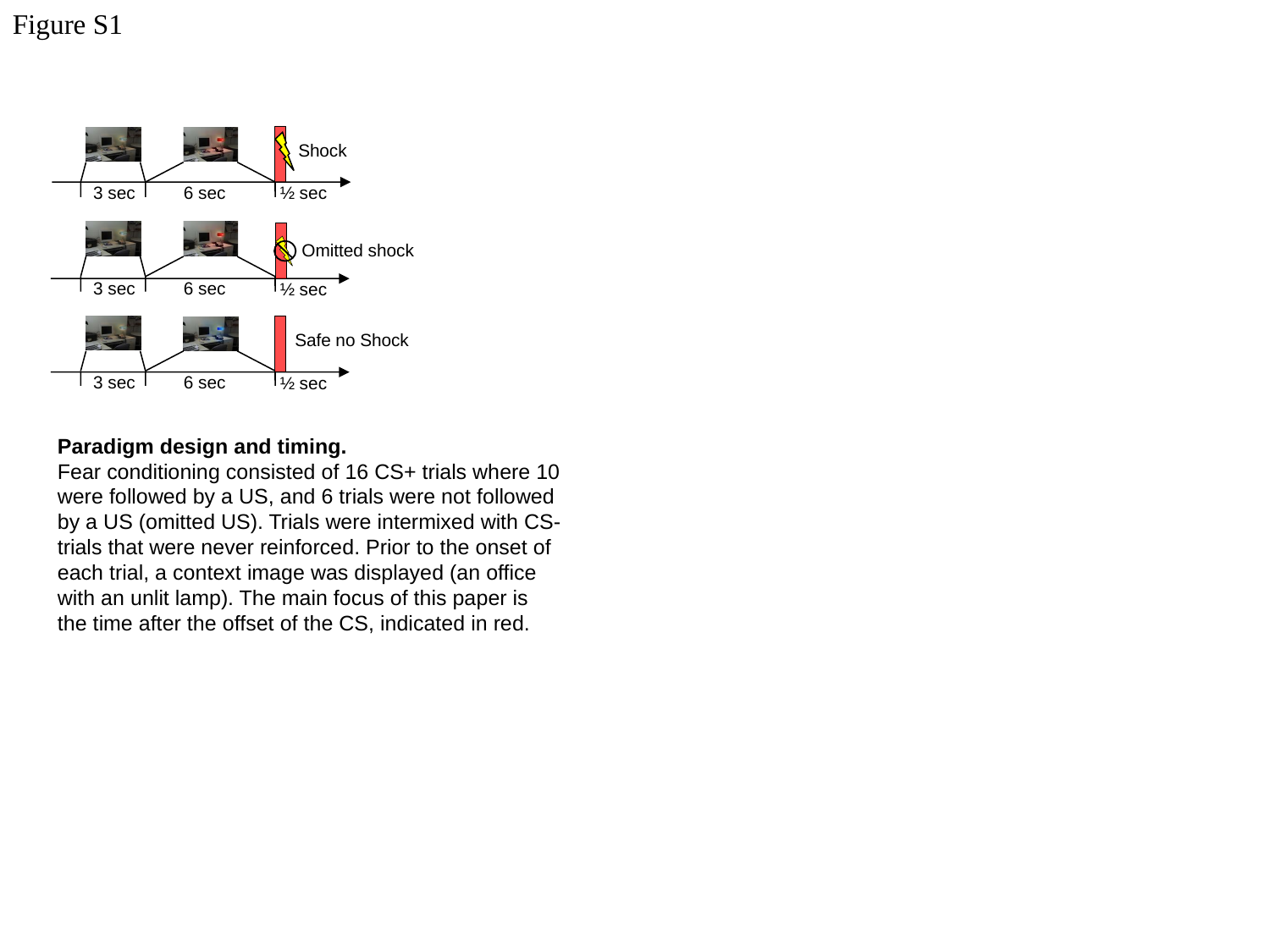

Figure S1
Shock
3 sec
6 sec
½ sec
Omitted shock
3 sec
6 sec
½ sec
Safe no Shock
3 sec
6 sec
½ sec
Paradigm design and timing.
Fear conditioning consisted of 16 CS+ trials where 10 were followed by a US, and 6 trials were not followed by a US (omitted US). Trials were intermixed with CS- trials that were never reinforced. Prior to the onset of each trial, a context image was displayed (an office with an unlit lamp). The main focus of this paper is the time after the offset of the CS, indicated in red.

## Slide 2
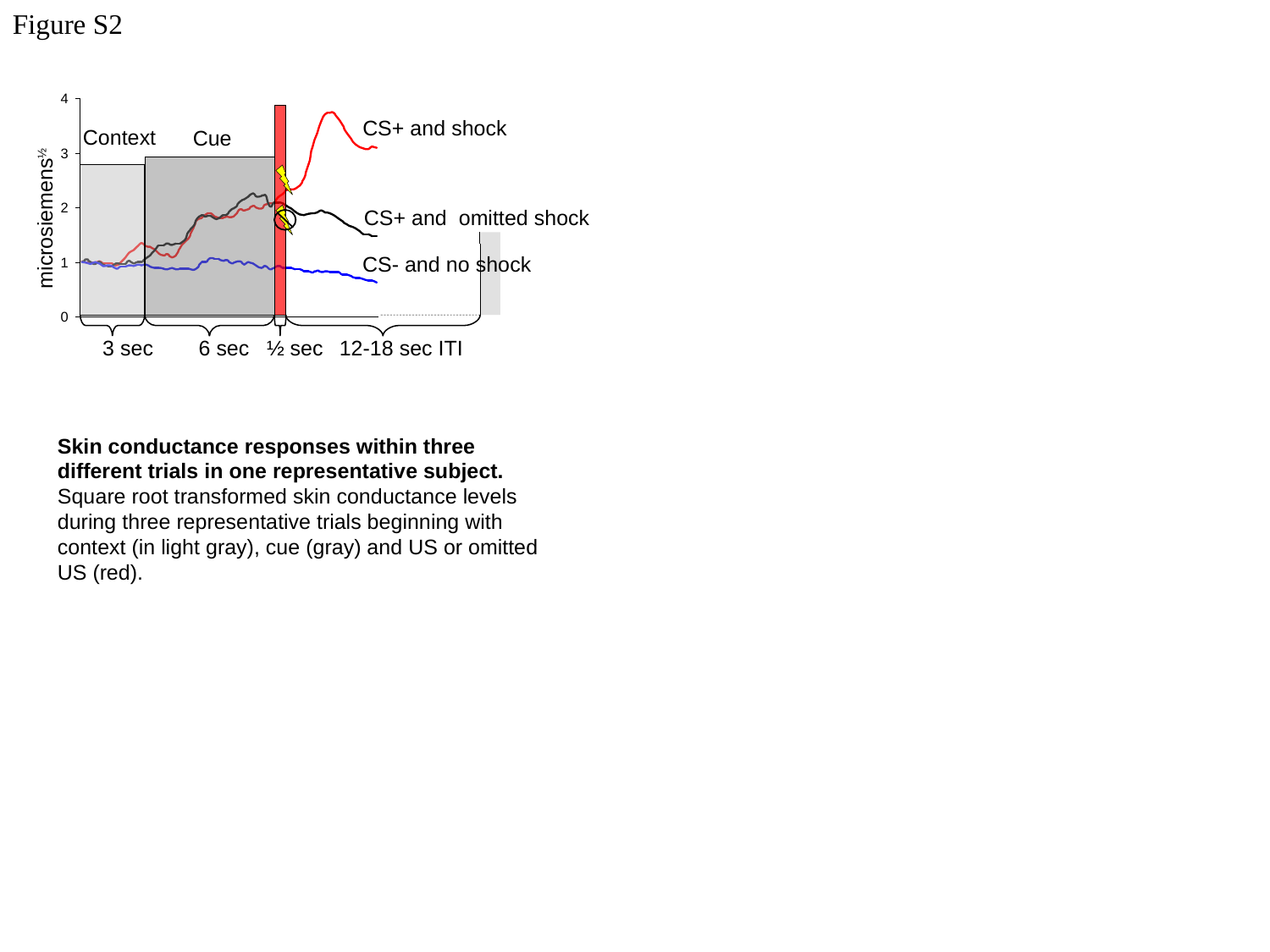

Figure S2
CS+ and shock
Context
Cue
CS+ and omitted shock
microsiemens½
CS- and no shock
3 sec
6 sec
½ sec
12-18 sec ITI
Skin conductance responses within three different trials in one representative subject.
Square root transformed skin conductance levels during three representative trials beginning with context (in light gray), cue (gray) and US or omitted US (red).

## Slide 3
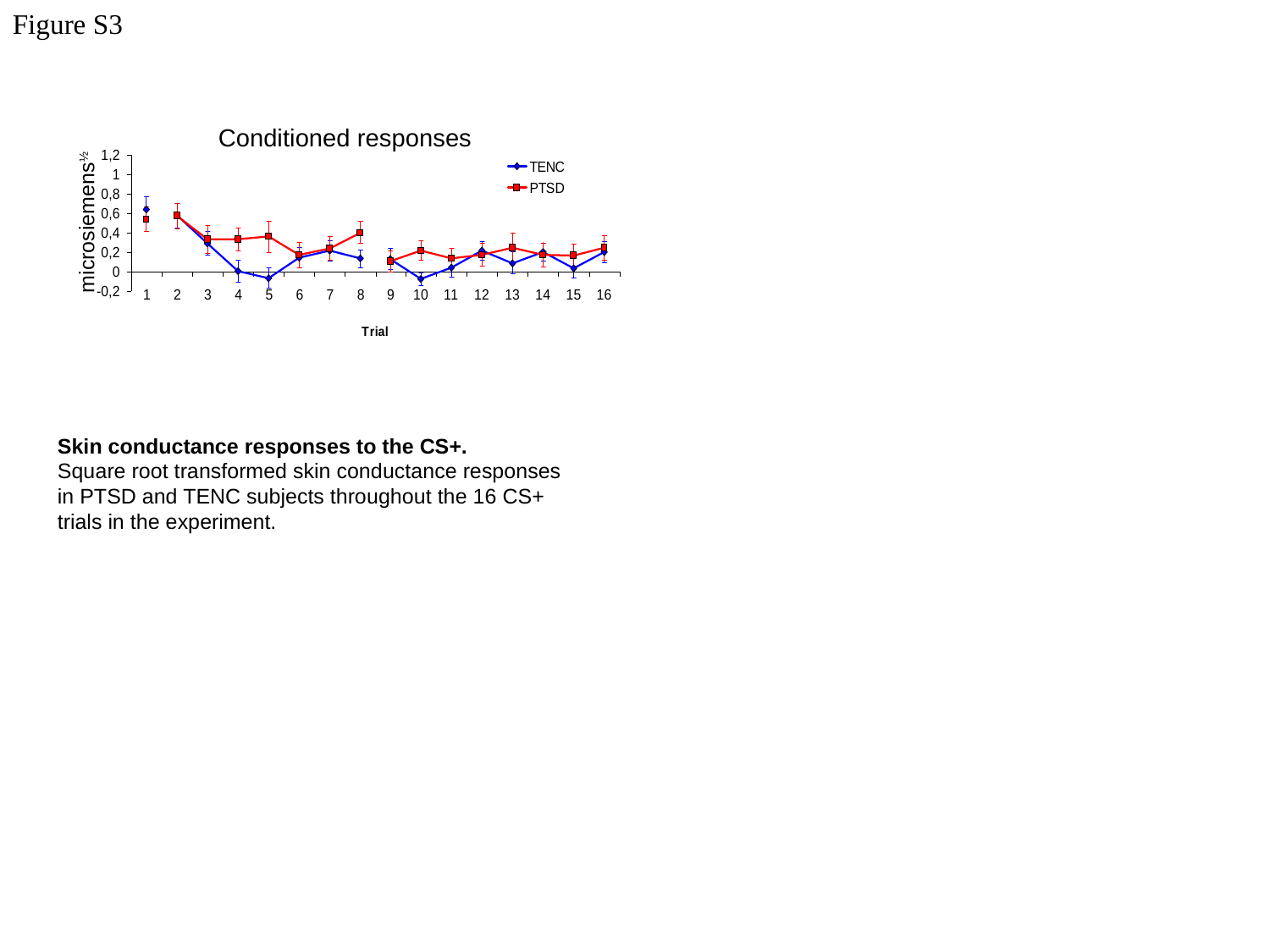

Figure S3
Conditioned responses
microsiemens½
Skin conductance responses to the CS+.
Square root transformed skin conductance responses in PTSD and TENC subjects throughout the 16 CS+ trials in the experiment.

## Slide 4
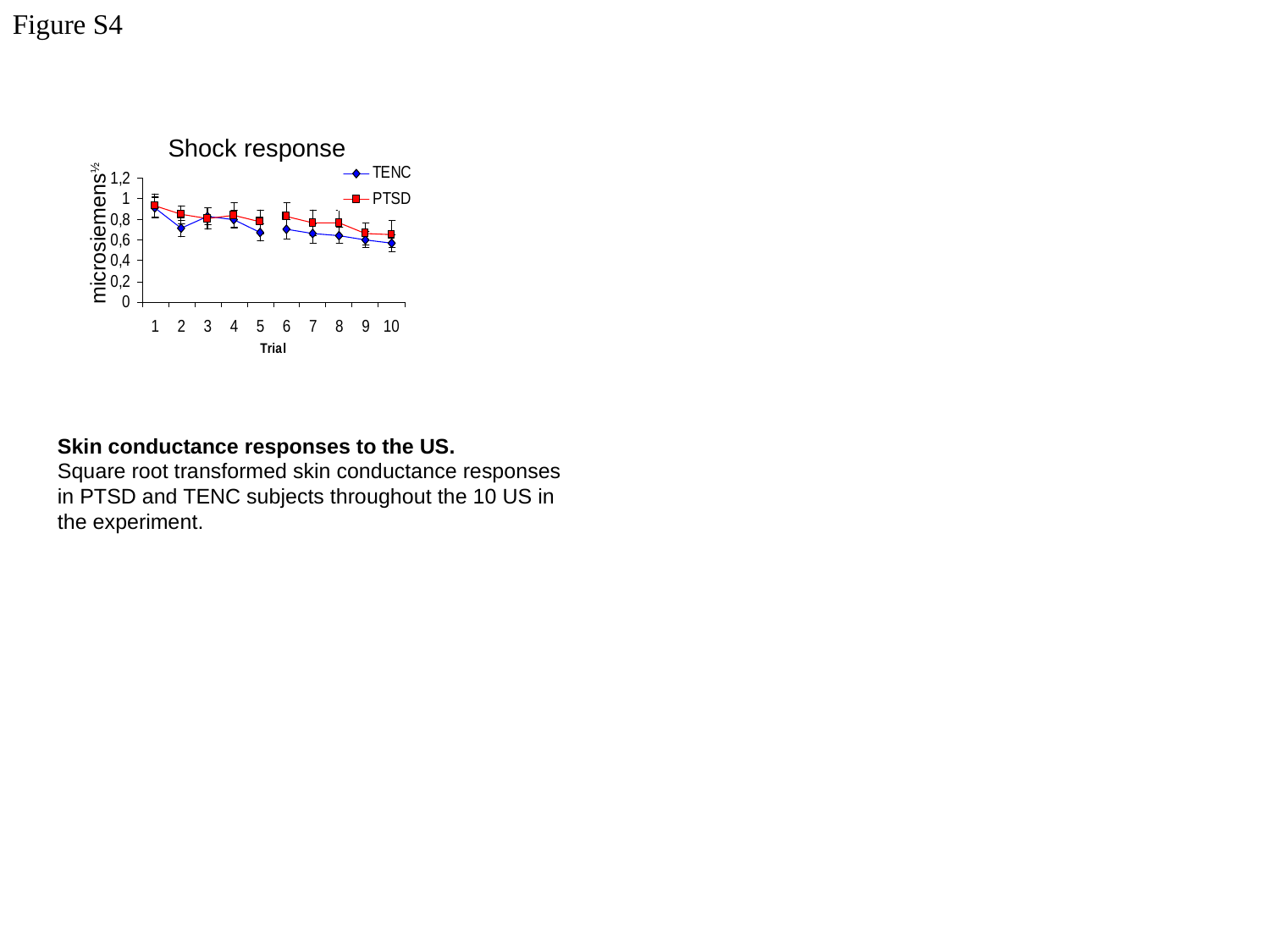

Figure S4
Shock response
microsiemens½
Skin conductance responses to the US.
Square root transformed skin conductance responses in PTSD and TENC subjects throughout the 10 US in the experiment.

## Slide 5
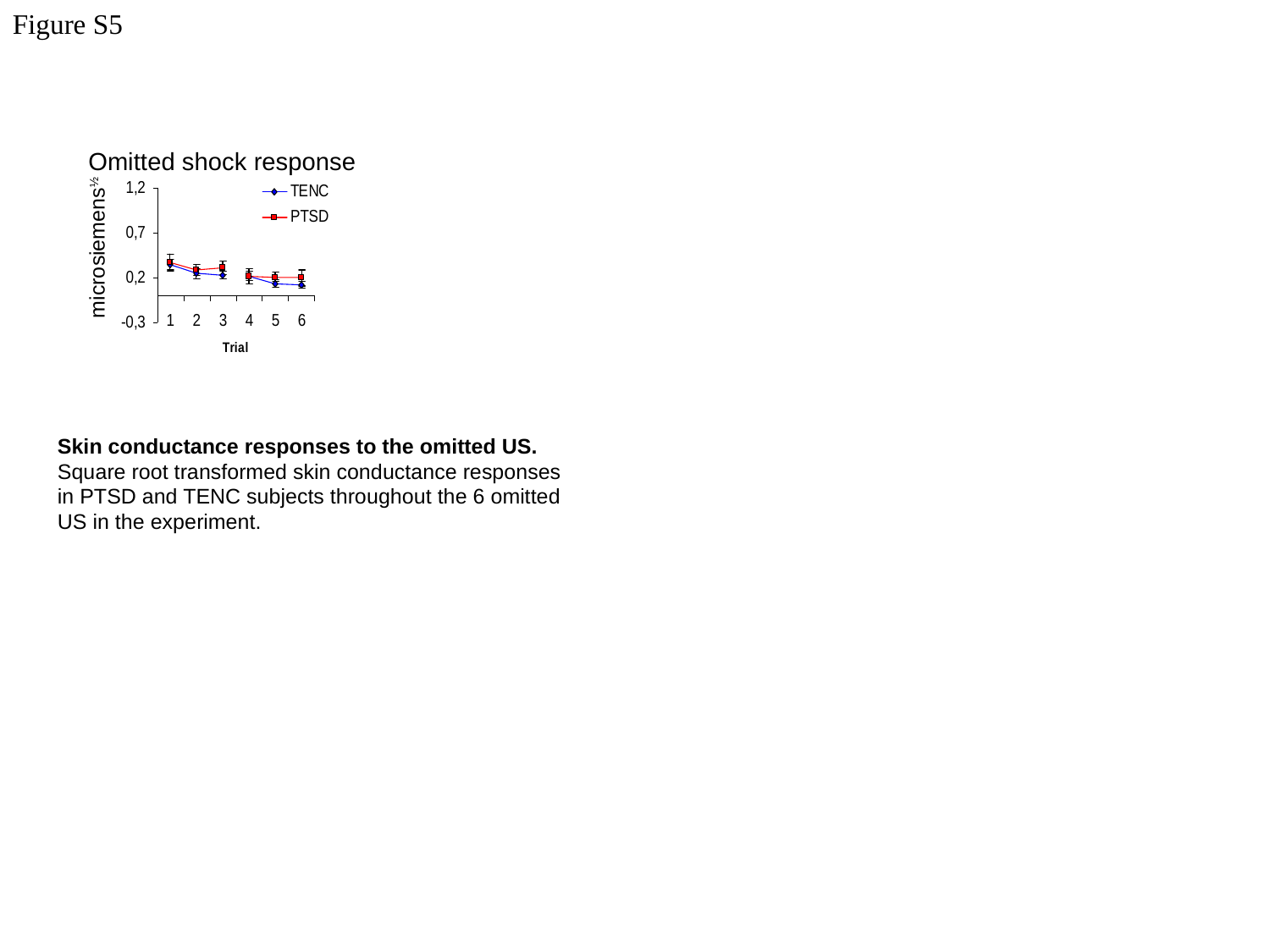

Figure S5
Omitted shock response
microsiemens½
Skin conductance responses to the omitted US.
Square root transformed skin conductance responses in PTSD and TENC subjects throughout the 6 omitted US in the experiment.
